# Causes of Disease in Large Cities

**Published:** 1874-09

**Authors:** 


### Editorial.

### Causes of Disease in large Cities.

Recently our attention has been called to the open privy-vaults, which by their foul emanations contaminate the atmosphere in many portions of our city, and are active and potent agents in the production of disease. These vaults are, in many instances unconnected with the sewers, and are not only open, but overflowing pools, and in most cases where there is such connection no arrangement is made to wash out the vault, or prevent the ingress of sewer gas.

Some means should be instituted whereby these active agents in the spread of disease may be abated.

If any one imagines that we are over fastidious in this matter, we will ask him to go with us to the upper rooms of some of the houses in this city, in the hot season, and we have no fear of his not being convinced of the near vicinity of one of these pests. It may be remarked that they are disagreeable but not dangerous. It can be plainly shown that they are highly dangerous to the public health. These privies are, many of them, placed in the yards of the houses to which they belong, some in the houses themselves; in these yards are in many instances wells from which the drinking water of the family is obtained, and in such close proximity are the wells that it requires no stretch of imagination to see how the contents of the privy vault might filter into the well. We know of one family, the members of which were sent into the country to recover from the effects of *nine open privy vaults within less than one hundred feet of their door.*

While upon this subject we desire to say a word or two in regard to the in-door water closets, which are used in many houses. These are convenient, and if properly constructed, safe substitutes for the privy, but too much care cannot be taken to prevent the entrance of sewer gas through improperly constructed or defective traps. Though elegant and convenient, they can be through the means of sewer gas the active agents of disease, and when physicians detect the slightest suspicion of its presence, they should at once warn their patients to look to their water-closet traps.

Sewer gas and the emanations from open cess-pools are active causes in the production of typhoid and other low forms of fever, and afford an explanation of the increased mortality of the city over the country.

